# Pedunculated pleomorphic adenoma of uvula – a rare presentation^☆^

**DOI:** 10.1016/j.bjorl.2020.12.013

**Published:** 2021-02-13

**Authors:** Sabarinath Vijayakumar, Shilpa Divakaran, Suhail Muzaffar, Mohammed Yusuf Mian, Irfan Khan

**Affiliations:** a Sandwell and West Birmingham NHS Trust, Birmingham, United Kingdom; b The Royal Wolverhampton NHS Trust, Wolverhampton, United Kingdom

## Introduction

Pleomorphic adenomas account for only about 6.5% of tumors in minor salivary glands, despite being the most common (70%) of all salivary gland neoplasms.^1^ The minor salivary glands neoplasms are relatively rare, with the palate as the most common site (66%), followed by buccal mucosa, lips and retromolar areas (1%).^2^

The uvula per se is a rare location for occurrence of such lesions, with only a few cases having been reported so far. The differential diagnosis includes squamous papilloma, other minor salivary gland tumors and epidermoid cyst, which commonly occurs in the pediatric age group.

## Case report

A 43 year old male presented with a short duration history of sudden choking sensation while eating, followed by a foreign body sensation in the throat for 2 weeks. He saw a swelling at the back of his throat, which prompted him to come to the hospital. He did not have any difficulty in breathing, although there was a slight discomfort while swallowing as well as snoring at night.

**Figure 1:**
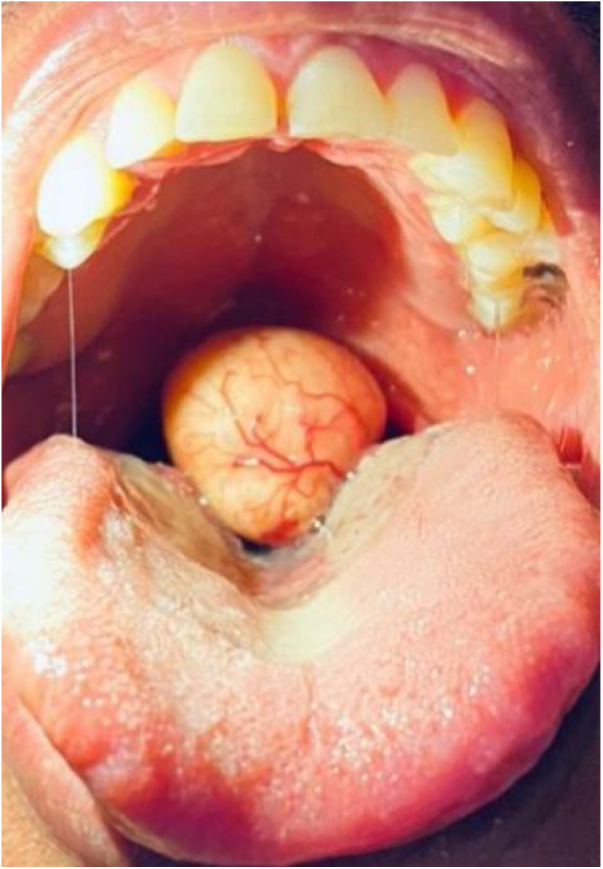
Intraoral appearance of the tumor.

On examination, he was comfortable at rest, with no signs of respiratory distress. There was a roughly 2 cm globular smooth pedunculated mucosa-covered mass from the posterior aspect of the tip of uvula, with overlying prominent vessels (Fig. 1). The remainder of the head and neck examination was unremarkable.

**Figure 2:**
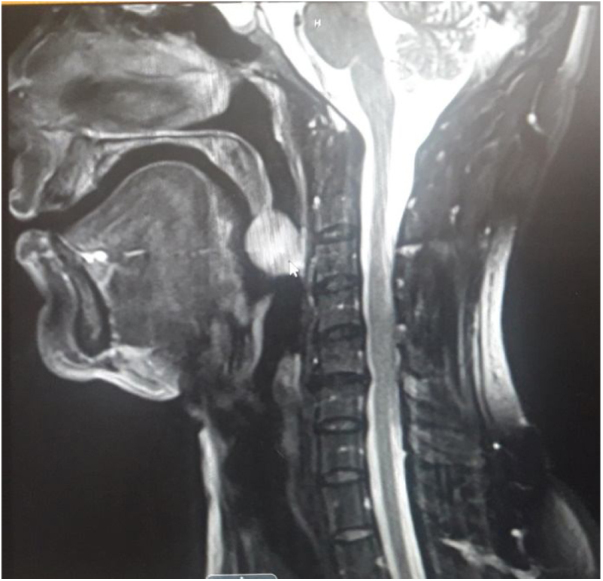
MRI showing ovoid enhancing mass lesion with well-defined margins hanging from tip of the uvula.

A magnetic resonance imaging (MRI) scan showed an ovoid enhancing mass lesion with well-defined margins hanging from tip of the uvula, measuring 2.3 × 2.1 × 2 cm (LS × TS × AP), without any invasion of surrounding tissues (Fig. 2).

Excision was performed under general anesthesia. In head extension with a mouth gag, using bipolar diathermy, the pedunculated mass was identified attached to posterior aspect of entire length of uvula and adjoining soft palate on the right side. Incision was created along the attachment and the mass excised in toto along with a part of uvula and soft palate; a palatoplasty was done to create a neo-uvula.

**Figure 3:**
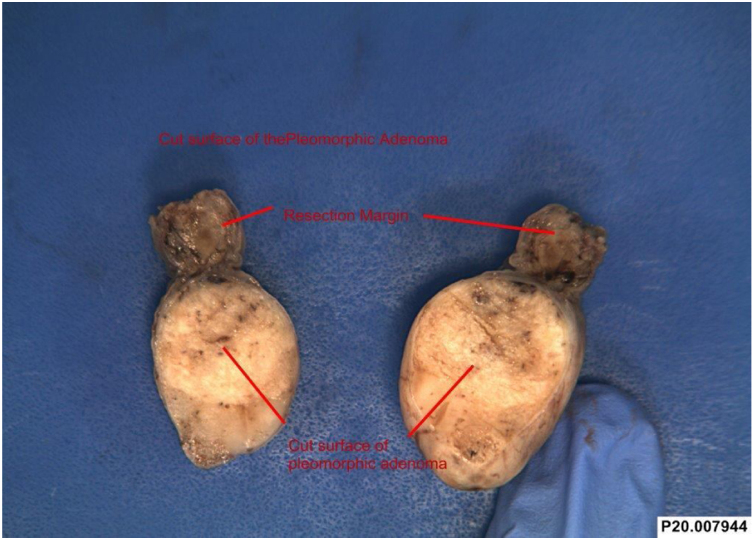
Excised tumor from the uvula.

On gross examination the tissue appeared to be a firm cream-colored intact rounded tissue fragment measuring 20 × 20 × 20 mm with attached light brown mucosa measuring 15 × 10 mm. Slicing revealed a firm, mucoid, cream colored cut surface (Fig. 3).

**Figure 4:**
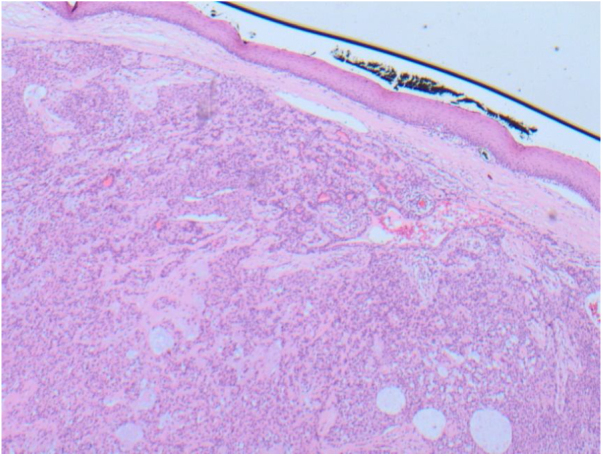
Histopathology of the excised lump showing a predominantly epithelial component arranged in tubules and cystic areas with myxomatous background, with palatal squamous epithelium above the lesion.

Microscopic examination of the lesion showed normal squamous mucosal lining epithelium containing minor salivary gland tissue which covered the entire surface of the lesion. A clear resection margin was present. The lesion was comprised of a well circumscribed benign mixed tumor composed of epithelial cells in sheets and cords embedded within a variably fibromyxoid stroma containing occasional myoepithelial cells. The epithelial cells were essentially monomorphic with plasmacytoid appearance. There was no evidence of nuclear atypia, mitoses, perineural or vascular invasion. These morphological features were those of a benign pleomorphic adenoma (benign mixed tumor). There was normal salivary gland tissue noted at the resection margin of the pedunculated lesion and therefore the lesion was considered completely excised (Fig. 4).

The patient was followed up and after one month, the palatal scar had healed well on examination. At three months and six months after the procedure, the patient was doing well, without any changes in speech, velo-pharyngeal insufficiency or tumor recurrence.

## Discussion

The most common benign tumor of the oral cavity is pleomorphic adenoma (50%), with the palate as the most common site. These tumors are seen between the fourth to sixth decade with a slightly female preponderance.^3^, ^4^ Histologically, these tumors have a mixed appearance of myoepithelial and epithelial components in a background of variable fibromyxoid stroma with a pseudocapsule.^5^

Pleomorphic adenomas are slow growing tumors, with patients presenting with long-standing symptoms over several months: dysphagia, sleep disturbances and snoring. There was one case reported by Fidan et al.^6^ of uvular pleomorphic adenoma presenting with otalgia, however that patient also had changes in voice. In this case we had a male patient with a short history of foreign body sensation for only 2 weeks.

Pleomorphic adenomas arising in the uvula are quite rare, with few cases reported to date.^6^, ^7^, ^8^ The differential diagnosis include squamous papilloma and epidermal cyst (which occurs mostly in the pediatric age group). In this case, the appearance was that of a smooth mucosa-covered firm swelling, which is unlike that of a papilloma or epidermoid cyst.

MRI scan is the investigation of choice in evaluating such soft tissue lesions as they provide good demarcation. Pleomorphic adenomas have low T1 – weighted signal and high T2 – weighted signal on MRI.^9^

Surgical excision is the preferred method of treatment. Pleomorphic adenomas, if incompletely excised, can result in recurrence.^10^ In this case, a cuff of normal tissue was also taken keeping in mind the probability of a salivary gland tumour. The remaining uvula was reconstructed to avoid speech and swallowing problems.

## Conclusion

Uvular lesions by themselves are quite uncommon. A possibility of minor salivary gland tumor should be kept in mind while encountering firm lesions of this area. Left untreated, these lesions can not only enlarge to cause significant morbidity in terms of airway and swallowing, but also carry a risk of malignant transformation.

## Ethical approval

Consent taken from the Institute and patient for publishing clinical photographs and writing the case report.

## Conflicts of interest

The authors declare no conflicts of interest.
